# Hand Tracking and Gesture Recognition Using Lensless Smart Sensors

**DOI:** 10.3390/s18092834

**Published:** 2018-08-28

**Authors:** Lizy Abraham, Andrea Urru, Niccolò Normani, Mariusz P. Wilk, Michael Walsh, Brendan O’Flynn

**Affiliations:** Micro and Nano Systems Centre of Tyndall National Institute, University College Cork, Cork T12 R5CP, Ireland; andrea.urru@upf.edu (A.U.); niccolo.normani@gmail.com (N.N.); mariusz.wilk@tyndall.ie (M.P.W.); michael.walsh@tyndall.ie (M.W.); brendan.oflynn@tyndall.ie (B.O.)

**Keywords:** LSS, Infrared LEDs, Calibration, Tracking, gestures, RMSE, Repeatability, Temporal Noise, latency

## Abstract

The Lensless Smart Sensor (LSS) developed by Rambus, Inc. is a low-power, low-cost visual sensing technology that captures information-rich optical data in a tiny form factor using a novel approach to optical sensing. The spiral gratings of LSS diffractive grating, coupled with sophisticated computational algorithms, allow point tracking down to millimeter-level accuracy. This work is focused on developing novel algorithms for the detection of multiple points and thereby enabling hand tracking and gesture recognition using the LSS. The algorithms are formulated based on geometrical and mathematical constraints around the placement of infrared light-emitting diodes (LEDs) on the hand. The developed techniques dynamically adapt the recognition and orientation of the hand and associated gestures. A detailed accuracy analysis for both hand tracking and gesture classification as a function of LED positions is conducted to validate the performance of the system. Our results indicate that the technology is a promising approach, as the current state-of-the-art focuses on human motion tracking that requires highly complex and expensive systems. A wearable, low-power, low-cost system could make a significant impact in this field, as it does not require complex hardware or additional sensors on the tracked segments.

## 1. Introduction

Three-dimensional (3D) ranging and hand tracking have a wide range of research applications in the field of computer vision, pattern recognition, and human–computer interaction. With the recent advancements of consumer-level virtual reality (VR) and augmented reality (AR) technologies, the ability to interact with the digital world using our hands gains significant importance. When compared to other body parts, tracking hand–finger movements is very challenging due to the fast motion, lack of visible textures, and severe self-occlusions of finger segments. The hand is one of the most complex and beautiful pieces of natural engineering in the human body. It gives us a powerful grip but also allows us to manipulate small objects with great precision. One reason for this versatility is its great flexibility, which allows it to move fast compared to other body parts such as legs or head, which is well explained in the work of Taylor et al. [1].

The evolution of hand tracking starts with conventional vision cameras. A detailed survey of various hand tracking methodologies and gesture recognition techniques using vision sensors are given in the works of Rautaray et al. [2]. The main drawback of vision cameras is their limitations in the two-dimensional (2D) domain, and in order to ensure consistent accuracy in tracking, it requires a focusing lens that makes the system bulky and expensive. Garg [3] used video cameras to recognize hand poses, ending in a highly complicated and inefficient process. Yang [4] suggested another method using vision sensors that also locate finger tips, but the method was not invariant to the orientation of the hand. Few other methods are dependent on specialized instruments and setup, such as the use of infrared cameras [5], and fixed background [6], which cannot be used in any environmental conditions. Stereoscopic camera systems make a good substitute for vision sensors, as they provide depth information about the scene, and thus the ability to reconstruct the hand in 3D in a more accurate fashion [7]. Yi-Ping et al. [8] suggested directionally variant templates to detect fingertips, which provide information about finger orientation. Some other works used the well-established Kalman filters together with depth data for more efficient tracking, leading to gesture recognition [9,10]. However, the uncontrolled environment, lighting conditions, skin color detection, rapid hand motion, and self-occlusions pose challenges to these algorithms when capturing and tracking the hand [11].

Over the past decades, a number of techniques based on glove technology have been explored to address the problem of hand–finger tracking. These can be categorized as data gloves, which use exclusive sensors [12], and marker-based gloves [13]. In some of the earlier works, the data gloves are constructed using accelerometers alone for the tracking [14,15], but in the recent works [16,17], data gloves are well equipped with inertial measurement units (IMUs) and magnetic field sensors that incorporate: an accelerometer, a gyroscope, and a magnetometer, whose combined outputs provide the exact orientation information. This allows for high dimensionality in terms of degrees of freedom, which indeed helps in tracking the movements of finger joints. Gloveone by Neuro Digital Technologies [18], VR Glove by Manus [19], and Noitom Hi5 VR Glove [20] are the predominant data gloves available in the market, which enable tracking all of the finger phalanges using a number of IMUs with low latency and often haptic feedback. Finger-joint tracking using hall-effect sensors [21] is another recently developed novel technology that provides higher joint resolutions than IMUs. However, the main drawback of these technologies is that they are not sufficient for providing exact location information, as the sensors are estimating only the orientation of the hands and fingers. Data gloves require complex algorithms to extract useful information from IMUs, and are not sufficient for providing exact location information. Instead of using sensors, gloves fitted with reflective markers or color patches are also developed, which provide a less complex solution than standard data capture gloves, but the device itself might inhibit free hand movements, and as such cannot be tracked precisely [22,23].

Recently, the various depth sensors in the market have become popular in the computer vision community exclusively for hand tracking and gesture recognition research activities. Leap Motion [24], Time-of-Flight (ToF) sensors [25], and Kinect depth sensors [26] are some of the commercially available depth sensors. The associated Software Development Kit (SDK) provided along with the sensors estimates the depth information [27,28,29] irrespective of the color values, which provides convenient information for gesture recognition [30].

The marker-less tracking of hands and fingers is another promising enabler for human–computer interaction. However, tracking inaccuracies, an incomplete coverage of motions, a low frame rate, complex camera setups, and high computational requirements are making it more inconvenient. Wang et al. [31] and Sridhar et al. [32] present remarkable works in this field, but with the expense of dense camera installations and high setup costs, which limits the deployment. Multi-camera imaging systems are also investigated that can estimate hand pose with high accuracy [33]. However, the calibration of multi-camera setups make these methods difficult to adopt for practical applications, as the natural hand movement involves fast motions with rapid changes and self-occlusions.

Most recently, new methods in the field of hand tracking and gesture recognition involve the utilization of a single depth camera as well as a fusion of inertial and depth sensors. Hand motion and position tracking are made possible by continuously processing 2D color and 3D depth images with very low latency. The approach uses strong priors and fails with complex self-occlusions, which often make it unsuitable for a real-time hand movement tracking, although it is widely accepted for tracking robotic trajectories [34]. The most recent and more predominant works in this field are by Sridhar et al. [35] and Tkach et al. [36], using either Intel Real Sense or Microsoft Kinect as a Red-Green-Blue-Depth (RGBD) camera. Another recent approach is the fusion of inertial and depth sensors [37,38], which is a practically feasible solution if very high accuracy in estimating 3D locations together with orientations are required.

In general, motion analysis using inertial measurement units (IMUs) requires complex hardware, as well as embedded algorithms. Conventional stereoscopic cameras or optical depth sensors used for high precision ranging are not suitable for a wearable system due to their high cost and impractical technical requirements. The other technologies that have been described require highly complex and expensive infrastructure. The applications of hand-tracking demand tracking across many camera-to-scene configurations, including desktop and wearable settings. As seen by the recent development of VR systems, such as the Oculus rift developed by Facebook, Google Glass by Google, or Microsoft HoloLens, the key requirement is the integration of mobility, low-power consumption, small form factor, and highly intelligent and adaptable sensor systems. Taking into account all of these constraints, this work is focused on the use of a novel Lensless Smart Sensor (LSS) developed by Rambus, Inc. [39]. The novel algorithms developed for this sensing technology allow the basic tracking of a number of infrared (IR) light-emitting diodes (LEDs) placed on key points of the hand, and thereby achieve the detection of both the position and orientation of the hand in real-time to millimeter-level accuracy with high precision at low cost. The tracking system requires reduced complexity hardware on the hand segments. As opposed to requiring significant infrastructural investment associated with the imaging cameras as the traditional approach to solving this problem, the hand-tracking methodology of this work uses light sensors only.

The paper is organized as follows: A brief description of Rambus’s LSS technology that is given in Section 2 and Section 3 describes the developed hand tracking and gesture recognition methodology in a systematic way. Both hand tracking and gesture recognition methods are validated in Section 4 using various experiments conducted with precise measurements. Finally, the work is concluded in Section 5.

## 2. Rambus Lensless Smart Sensor

Lensless Smart Sensors (LSS) stemmed from a fundamental question of what could be the smallest optical element that can be used to sense. The traditional optical lens is usually the limiting factor, in terms of defining how small the imaging device can be. Rambus pioneered a near field diffraction grating (patents issued and pending) that creates predictable light patterns on the sensor. Thus, the lens used in a conventional vision sensor is replaced by a spiral-shaped diffraction grating. The 1-mm thick LSS ensures a very small form factor for a miniaturized, wearable tracking system. The unique diffraction pattern associated with the LSS enables more precise position tracking than possible with a lens by capturing more information about the scene. However, instead of getting the image as such, the sensors will only provide unclear blob-like structures [40] that are rich in information. A proper reconstruction algorithm is developed using the blob data and the knowledge of grating to reconstruct the image without losing accuracy, which is well explained in previous works [41,42]. The sensing technology, as well as the optical, mathematical, and computational foundations of the LSS, are discussed in detail in the works of Stroke [43] and Gill [44].

## 3. Methodology

In order to make the system usable in a wide range of environmental conditions, infrared LEDs have been used in the tracking system, as they are impervious to white light sources in standard daylight conditions, when using the LSS in combination with an infrared filter. The method to range and track a single light source is well described in previous work [41,42]. The work described in this paper is focused on developing a method for tracking multiple light points simultaneously, and thereby estimating hand location and orientation; thus, enabling smart tracking of a hand.

### 3.1. Physical Setup

**Figure 1:**
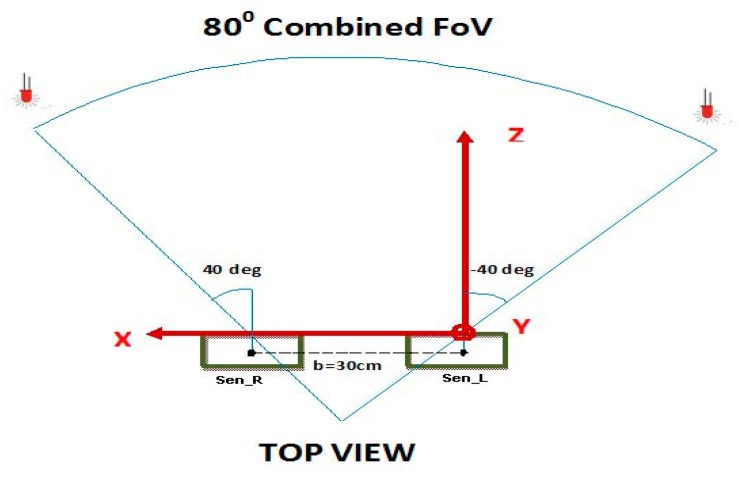
System overview.

The stereoscopic field of vision (FoV) is defined on the basis of the following parameters (Figure 1):

(i) the longitudinal distance along the *Z*-axis between the LED and the sensor plane goes from 40 cm to 100 cm;
(ii) the distance between the right and left sensors (*Sen_R* and *Sen_L* respectively, as shown in Figure 1) measured on the central points of the sensors, called baseline (*b*) is 30 cm;
(iii) the combined FoV of 80°.

The work envelope is defined in this way since the typical range of motion for hand tracking and gesture recognition applications is confined more or less within these constraints [2].

### 3.2. Constraints for Multiple Points Tracking

The first stage of the experimental setup was an initial validation phase; a feasibility study was carried out by tracking two points simultaneously to understand the possible issues that can occur when dealing with tracking multiple points of light. Based on this initial analysis, two constraints are observed:

(i) Discrimination—When the distance between two light sources along the *X*- (or *Y*-) axis is less than 2 cm, the two sources will merge to a single point in the image frame, which makes them undistinguishable.

**Figure 2:**
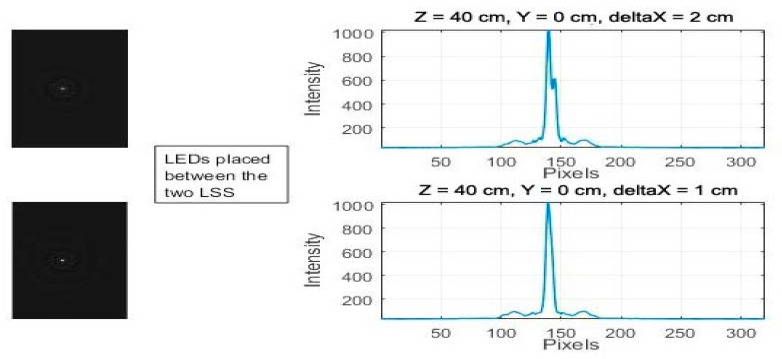
Light-emitting diodes (LEDs) placed at *Z* = 40 cm moved closer from 2 cm and 1 cm.

**Figure 3:**
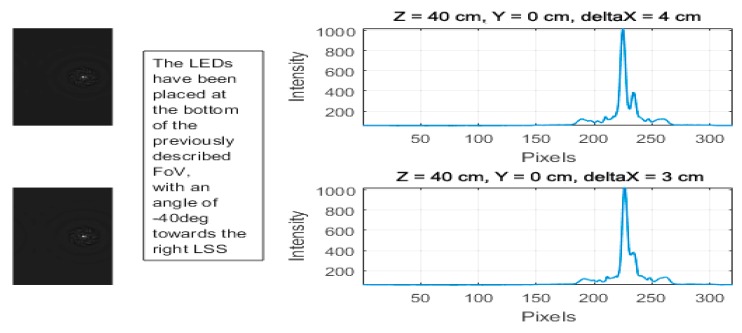
LEDs placed at FoV = 40° moved closer from 4 cm to 3 cm.

In the graph below (Figure 2), the two LEDs are progressively moved closer to each other, from 2 cm to 1 cm along the *X*-axis, at a distance of *Z* = 40 cm and *Y* = 0 cm. Both LEDs are in a central position between the two LSSs, and it is seen that the two light sources merge into a single point that can no longer be distinguished.

(ii) Occlusion—Occlusions occur when the light source is moved away from the sensor focal point along the *X*-axis. In Figure 3, at extreme lateral positions when the FoV ≥ 40°, even when the LEDs are 3 cm away, one LED is occluded by the other.

### 3.3. Placement of Light Points

**Figure 4:**
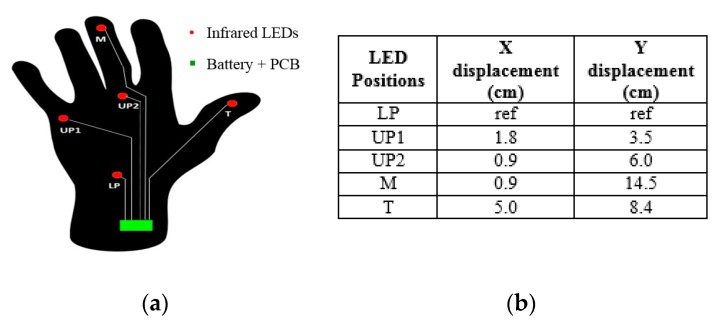
(**a**) Placement of LEDs in hand; (**b**) *X* and *Y* displacement of LEDs.

The LEDs are placed on the hand in such a way as to minimize both the discrimination/occlusions problems, and the number of LEDs adopted, yet still allowing the recognition of the hand. Thus, three LEDs are used on the palm with the maximal possible distance between them (LP—Lower Palm, UP1—Upper Palm 1, UP2—Upper Palm 2). In this scenario, the palm is considered a flat surface to make the model simple for the first basic version of tracking using the LSS. The main objective was to make a workable system using light sensors alone, taking account of more constraints in the next versions by combing the light sensors with other possible sensors. It was important, because at least three points are required to estimate the orientation of a plane, which is how the hand orientation is determined. In addition, one LED is used in the middle finger (M) just above the upper palm LED to track the movement that is assigned to all of the other fingers, except for the thumb, to prove the concept of finger/multiple segment tracking. An additional LED is placed on the thumb (T) to track its movement. Figure 4a shows the positional overview of LEDs on the hand. Taking *LP* LED as a reference, the *X* and *Y* displacements of all of the other LEDs are calculated (Figure 4b).

### 3.4. Hardware Setup

**Figure 5:**
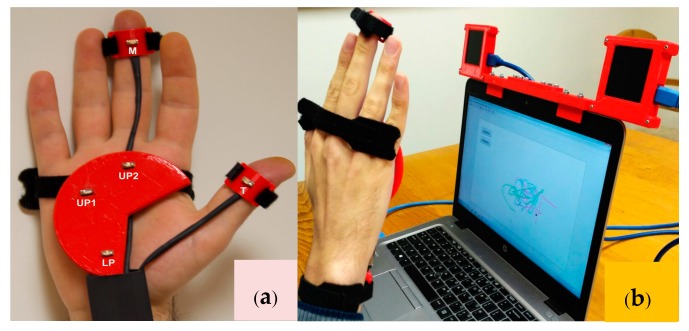
(**a**) Setup for hand; (**b**) final setup for hand tracking.

The experimental setup fixture consists of a 3D printed holder that is designed to hold five LEDs in the prescribed locations on the hand. This is then secured to the user’s hand through simple straps, as shown in Figure 5a. The LSSs are placed in 3D-printed casings with the infrared filters [42] attached, as seen in the final setup shown in Figure 5b. It allows for an embedded platform that can be easily mounted on top of any laptop display. Since the sensing is based on diffraction optics, it is necessary to ensure that the LSSs are both carefully aligned on a common plane with no possible undesired rotations or translations while fixing them inside the enclosures.

### 3.5. Multiple Points Tracking

The single point-tracking algorithm can be extended to a multiple points tracking scenario. As the sensors are not providing the hand image as such, and no IMUs are fitted on the hand, only the 3D LED locations are obtained. Therefore, the multiple points tracking methodology is formulated in a different way, utilizing the basic ideas of Extended Kalman filter tracking, and Random Sample Consensus (RANSAC) algorithms from the target-tracking works of [45,46,47]. The algorithm can be structured into two main phases, namely: calibration and tracking.

#### 3.5.1. Calibration Phase

**Figure 6:**
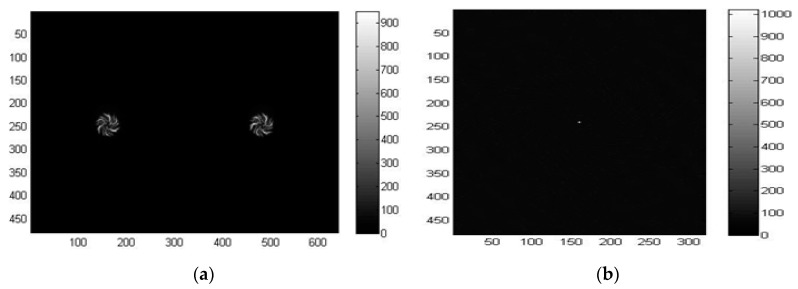
(**a**) Reference point-spread function (PSF); (**b**) reconstructed point.

In this phase, the hand must be kept stationary in front of the sensors, with the palm and fingers open, and the middle finger pointing upwards. The calibration is performed only once to save the relative distances of LEDs, and use them as a reference in the next phase of tracking. The raw images are captured and reconstructed according to the method explained in [41]. In short, the spiral-shaped diffractive grating of the LSS creates equally spaced dual spirals for a single point source of the light, as shown in Figure 6a. This is taken as the reference point-spread function (PSF) for the LSS. The regularized inverse of the Fourier transformation of the PSF, which is also called the kernel, is then calculated. The inverse Fourier transform of the product between the kernel and the raw image obtained from the LSS, which is the dual spiral image itself for a point source of light, is the final reconstructed image. It is seen from Figure 6b that the point source of light is reconstructed with low noise from the dual spiral pattern provided by the LSS. The two spirals on the dual gratings are not identical to each other; indeed, one is rotated 22° with respect to the other so that one spiral could cover the potential deficiencies present in the other sensor, and vice versa. Therefore, the reconstruction result for each spiral is used separately and then added together. This also allows effectively doubling the amount of light captured, thus reducing noise effects.

The algorithm then looks for as many points as specified as the inputs (three for the palm, two for fingers) by searching the local maxima. The maxima detection algorithm starts with the highest threshold (*th =* 1) and iteratively decreases the threshold for each image frame until all five LEDs are detected correctly by both sensors. The lowest threshold is saved as the reference (*th_ref_*) for the tracking phase. When the five detected points are correctly identified during the process, every point is labeled in order to assign each to the right part of the hand. The reconstructed frames have their origin in the top-left corner, with a resolution of 320 × 480, which is half the size along the *X*-direction and the full size along the *Y*-direction compared to the original image frames, as shown in Figure 6b.

Consequently, the following a priori information is considered for labeling based on the positioning of LEDs in the hand (Figure 5a):

- The middle finger point (*M*) has the lowest row coordinate in both images.
- The lower palm point (*LP*) has the highest row coordinate in both images.
- The first upper palm point (*UP*1) has the lowest column coordinate in both images.
- The second upper palm point (*UP*2) has the second column coordinate in both images.
- The thumb point (*T*) has the highest column coordinate in both images.

The 2D coordinates in the image domain, relative to the two sensors together with the assigned labels, are processed according to the point tracking algorithm [41,42], using the reference that the PSF created. Thus, a matrix of relative distances of the palm coordinates is computed, as given in Table 1 using Equation (1). This unique measure together with the reference threshold will be used in the following phase to understand the configuration of the hand.

(1) $$d_{p_{i},p_{j}}=\sqrt{{\left(x_{p_{i}}-x_{p_{j}}\right)}^{2}+{\left(y_{p_{i}}-y_{p_{j}}\right)}^{2}+{\left(z_{p_{i}}-z_{p_{j}}\right)}^{2}},\;\;p_{i},p_{j}=LP,\;UP1,\;UP2$$

#### 3.5.2. Tracking Phase

During the tracking phase also, the raw images from the left and right sensors are captured and reconstructed according to the procedure explained in [39], which is briefly described in the calibration phase. The algorithm then looks for local maxima that lie above the reference threshold *th_ref_*, which was saved during the calibration phase. The tracking phase is developed in such a way that it is impossible to start tracking if at least three LEDs are not visible for the first 10 frames, and the number of points in the left and right frames is not equal, in order to always maintain a consistent accuracy. As such, if *mL* and *mR* represent the detected maxima for *sen_L* and *sen_R* respectively, the following different decisions are taken according to the explored image:

- |*mL*| ≠ |*mR*| and |*mL*|, |*mR*| > 3: a matrix of zeros is saved and a *flag* = 0 is set encoding “Unsuccessful Detection”.
- |*mL*|, |*mR*| < 3: a matrix of zeros is saved and a *flag* = 0 is set encoding “Unsuccessful Detection”.
- |*mL*| = |*mR*| and |*mL*|, |*mR*| > 3: the points are correctly identified, *flag* = 1 is set encoding “Successful Detection”, and the 2D coordinates of LED positions are saved.

(a) Unsuccessful Detection (*Flag =* 0)

In this case, the useful information to provide an estimate of the current coordinates is determined using a polynomial fit function based on the 10 previous positions of each LED. The current coordinates to be estimated are defined as in Equation (2):

[*x*_0,*i*_, *y*_0,*i*_, *z*_0,*i*_] *i* = *M*, *LP*, *T*, *UP*1, *UP*2 (2)

At each iteration, the progressive time stamp of the 10 previous iterations (3) and tracked 3D coordinates of each axis relative to the 10 iterations (4) are saved.

[*t*_−10_ … *t*_−1_] (3)

{[*x*_−10,*i*_, …, *x*_−1,*i*_], [*y*_−10,*i*_, …, *y*_−1,*i*_], [*z*_−10,*i*_, …, *z*_−1,*i*_]} (4)

The saved quantities are passed to a polynomial function, as shown in Equation (5):

*f*_*poly*_*p*,*j*__ = *a*_*p*,*i*_*t*^2^ + *b*_*p*,*i*_*t* + *c*_*p*,*i*_, *p* = [*x*, *y*, *z*] (5)

The polynomial function performs the fitting using the least squares method, as defined in Equation (6):

(6) $$\left[{\hat{a}}_{p,i},{\hat{b}}_{p,i},{\hat{c}}_{p,i}\right]={\mathrm{argmin}}_{a_{p,i},b_{p,i},c_{p,i}}\;\overset{-1}{\underset{j=-10\;}{\sum }}{\left(p_{j,i}-a_{p,i}t_{j}{}^{2}+b_{p,i}t_{j}+c_{p,i}\right)}^{2}$$

Thus, the function *f_poly_* estimates the coordinates of all five LED positions related to the current iteration, based on the last 10 iterations, as shown in Equation (7):

(7) $$p_{0,i}={\hat{a}}_{p,i}t_{0}{}^{2}+{\hat{b}}_{p,i}t_{0}+{\hat{c}}_{p,i}$$

(b) Successful Detection (*Flag* = 1)

In this case, the coordinates are preprocessed by a sub-pixel level point detection algorithm [42,48], which enhances the accuracy of peak detection by overcoming the limitation of the pixel resolution of the imaging sensor. The method will avoid many worst case scenarios, such as the striking of the LEDs on the sensors being too close to one another and the column coordinates of different points perhaps sharing the same value, ending in a wrong 3D ranging. The matrix of coordinates can then be used to perform the labeling according to the proposed method, as described below:

(i) Labeling Palm Coordinates

Given *m_i_*, where *i* = *L*, *R*, as the number of maxima detected by both sensors, the combinations without repetitions of three previously ranged points are computed. Indeed, if five points are detected, there will be 10 combinations: four combinations for four detected points, and one combination for three detected points. Thus, triple combinations of *C* points are generated. The matrix of relative distances for each candidate combination of points, as *k* ∈ [1, …, |C|], is calculated using Equation (1). The sum of squares of relative distances for each matrix is then determined by Equations (8) and (9) for both the calibration and tracking Phases, as *k* ∈ [1, …, |C|]:

(8) $$[S_{d_{p_{i},p_{j}\;}}(k)]=\sum_{p_{i},p_{j}\in ck}d_{p_{i},p_{j}}^{2}$$

(9) $$[Sref]=\sum_{p_{i},p_{j}\in ref\;}d_{p_{i},p_{j}}^{2}$$

The results are summed again to obtain a scalar, which is the measure of the distance between the points in the tracking phase and the calibration phase, as shown in Equations (10) and (11), respectively:

(10) $$Sum_{k}=\sum \left[S_{d_{p_{i},p_{j}}}\right]$$

(11) $${Sum}_{ref}=\sum \left[S_{ref}\right]$$

where both $[S_{d_{p_{i},p_{j}}}]\in {\mathbb{R}}^{3}$ and $[S_{ref}]\in {\mathbb{R}}^{3}$ are vectors.

The minimum difference between the quantities *Sum_k_* and *Sum_ref_* can be associated with the palm coordinates. To avoid inaccurate results caused by environmental noise and possible failures in the local maxima detection, this difference is compared to a threshold to make sure that the estimate is sufficiently accurate. Several trials and experiments were carried out to find a suitable threshold to the value *th_distance_* = 30 cm. The closest candidate $\hat{k}$ was then found using Equation (12):

(12) $$\hat{k}=argmin_{k\in c_{k}}\left|Sum_{k}-Sum_{ref}\right|<th_{distance}$$

The final step was to compute all of the permutations of the matrix of relative distances ${\left[S_{d_{p_{i},p_{j}}}\left(\hat{k}\right)\right]}_{perm}$, and the corresponding residuals with respect to the reference matrix $\left[S_{ref}\right]$ (13):

(13) $$Res_{perm}=\left[S_{d_{p_{i},p_{j}}}{\left(\hat{k}\right)}_{perm}-\left[S_{ref}\right]\right]\forall \mathrm{permutations}$$

The permutation, which shows the minimum residual, corresponds to the labeled triple of the 3D palm coordinates. The appropriate labels are assigned to each of the points, as follows: $X_{LP1}$, $X_{UP1}$, $X_{UP2}$.

(ii) Labeling Finger Coordinates

After identifying the palm coordinates, the fingers that are not occluded are identified using their positions with respect to the palm plane by knowing their length and the position of the fingertips. The plane identified by the detected palm coordinates *X_plane_* is the normal vector to the palm plane. It is calculated using the normalized cross-product of the 3D palm coordinates of the points using the right-hand rule, as in Equation (14):

(14) $$X_{plane}=\frac{\left(X_{UP1}-X_{LP1})\times (X_{UP2}-X_{LP1}\right)}{\left|\left(X_{UP1}-X_{LP1})\times (X_{UP2}-X_{LP1}\right)\right|}$$

The projections of the unlabeled LEDs onto the palm plane (if there are any) are computed based on the two possible cases:

*Case* 1—*Two Unlabeled LEDs*:

The projected vectors for unlabeled LEDs *UL*_1_ and *UL*_2_ onto the palm plane are calculated by finding the inner product of *X_plane_* and ($X_{UL_{i}}$ − $X_{UP2}$). It is then multiplied by *X_plane_* and subtracted from the unlabeled LED positions, as shown in Equation (15):

(15) $$Proj_{UL_{i}}=X_{UL_{i}}-\left[X_{plane}\left(X_{UL_{i}}-X_{UP2}\right)\right]\;X_{plane},\;i=1,2$$

Two candidate segments $X_{UL_{1}}$ and $X_{UL_{2}}$ are then calculated as shown in Equations (16) and (17) and compared to the reference palm segment defined by $X_{ref}$ in Equation (18):

(16) $$X_{UL_{1}}=Proj_{UL_{1}}-X_{UL_{2}}$$

(17) $$X_{UL_{2}}=Proj_{UL_{2}}-X_{UP2}$$

(18) $$X_{ref}=X_{UP2}-X_{LP}$$

The results are used for the calculation of angles between them, as shown in Equations (19) and (20):

(19) $$\theta_{UL_{1}}=arccos\left(\frac{X_{UL_{1}}\cdot X_{ref}}{\left|X_{UL_{1}}\right|\left|X_{ref}\right|}\right)$$

(20) $$\theta_{UL_{2}\;}=arccos\left(\frac{X_{UL_{2}}\cdot X_{ref}}{\left|X_{UL_{2}}\right|\left|X_{ref}\right|}\right)$$

The middle finger is selected as the unlabeled LED *UL_i_*, which exposes the minimum angle, while the thumb is selected as the maximum one, as shown in Equations (21) and (22):

(21) $$X_{T}=argmax_{UL_{1}}{,}_{UL_{2}}[\theta_{UL_{1}},\theta_{UL_{2}}]$$

(22) $$X_{M}=argmin_{UL_{1}}{,}_{UL_{2}}[\theta_{UL_{1}},\theta_{UL_{2}}]$$

*Case* 2—*One Unlabeled LED*:

If only one of them is visible, the single projected vector is used to compute the angle between the segment *X_UL_* and the segment *X_ref_* in the same way, as shown in Equation (19)*.* The decision is made according to an empirically designed threshold *th_angle_* = 30°, as shown in Equation (23):

(23) $$UL=\{\begin{array}{c} X_{M},\;if\;\theta_{UL}\leq 30{}^{\circ}\\ X_{T},\;if\;\theta_{UL}>30{}^{\circ} \end{array}$$

(c) Occlusion Analysis

When the flag is set to 1, but one of the LEDs is occluded, its position is predicted using the method given in Section 3.5.2(a). The possibility of two different cases are as follows:

*Case* 1—*Occluded Finger*:

In this case, if there is no occlusion in the previous frame, the procedure is same as that of Section 3.5.2(ii)*.* If a previous frame has occlusion, the coordinate is chosen according to the coordinate $X_{UP2}$.

*Case* 2—*Occluded Palm*:

If none of the candidate combinations satisfy the *th_distance_* constraint given in Equation (12), proceed in the same way as that of Section 3.5.2(a). Thus, the proposed novel multiple points live tracking algorithm labels and tracks all of the LEDs placed on the hand.

#### 3.5.3. Orientation Estimation

**Figure 7:**
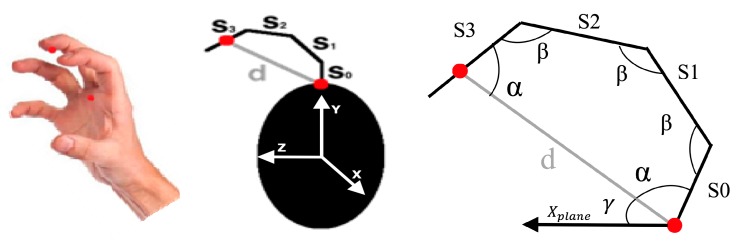
Modeling of palm to middle finger orientation.

Although the proposed tracking technique has no other sensors fitted on the hand to provide the angle information, and the tracking based on light sensors is able to provide only the absolute positions, the hand and finger orientations can be estimated. The orientation of the hand is computed by finding the plane identified by the palm *X_plane_* in Equation (14) and the direction given by the middle finger. If the palm orientation, the distance between initial and final segments (*S*_0_ and *S*_3_) of middle finger (*d*), and the segment lengths ($S_{il}$, *i =* 0,1,2,3) are assigned as known variables, the orientation of all of the segments can be estimated using a pentagon approximation model, as shown in Figure 7.

For this purpose, the angle γ between the normal vector exiting the palm plane *X_plane_*, as previously calculated in Equation (14), and the middle finger segment ($X_{UP2}$ − *X_M_*), is calculated as shown in Equation (24). The angle between *S*_0_ and *d* (=*α*) is its complementary angle is shown in Equation (25).

(24) $$\gamma =arcos\left(\frac{\left(X_{M}-X_{UP2\;}\right)\cdot X_{plane}}{\left|X_{M}-X_{UP2}\right|\left|X_{plane}\right|}\right)$$

(25) α=90°−γ

For the approximation model, it is assumed that the angle between *S*_0_ and *d* is equal to the angle between *S*_3_ and *d*. Thus, knowing that the sum of the internal angles of a pentagon is 540°, the value of the other angles (=*β*) is also computed assuming that they are equal to each other, as shown in Equation (26).

(26) β=(540−2×α)/3

The vector perpendicular to the middle finger segment and *X_plane_* is then computed as shown in Equation (27), and the rotation matrix *R* is built using angle *α* around the derived vector according to Euler’s rotation theorem [49]. This rotation matrix is used to calculate the orientation of segment S_0_ in Equation (28) and its corresponding 2D location in Equation (29).

(27) $$X_{⊥(plane↔M)}=\frac{X_{plane}\times (X_{M}-X_{UP2})}{\left|X_{plane}\times (X_{M}-X_{UP2})\right|}$$

(28) $$S_{0↗}=R\times \left(\frac{\left(X_{M}-X_{UP2}\right)}{\left|X_{M}-X_{UP2}\right|}\right)$$

(29) $$X_{S_{0}}=X_{UP2}+S_{0↗}\times S_{0l}$$

Thus, the orientation of all of the phalanges (*S*_1_, *S*_2_, and *S*_3_), as well as their 2D locations, is calculated. The same method is applied for finding the orientation of all of the thumb segments using the segment lengths and 2D location of lower palm LED (*X_LP_*), but with a trapezoid approximation for estimating the angles.

### 3.6. 3D Rendering

**Figure 8:**
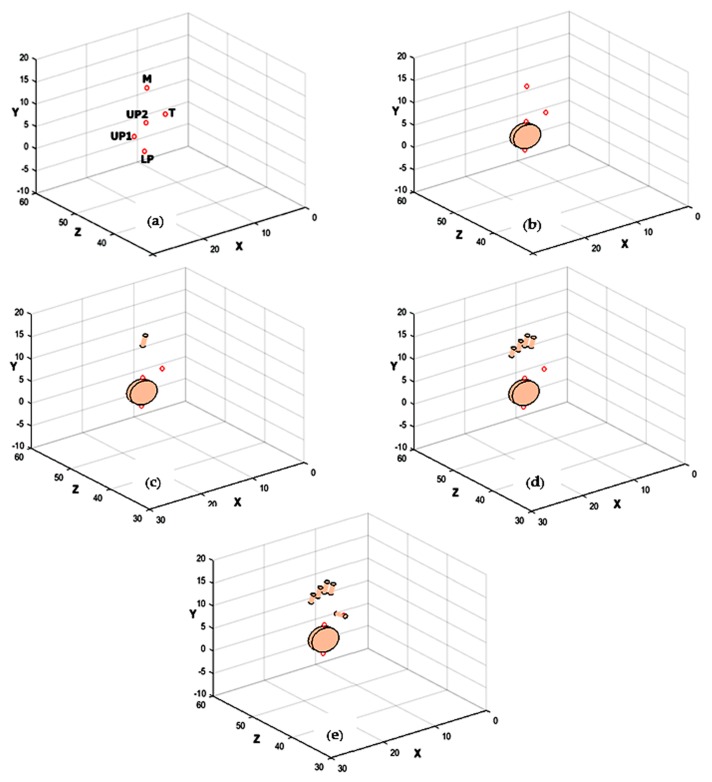
3D Rendering of hand (**a**); calculated LED positions (**b**); palm plane reconstructed using Three LEDs on Palm (*LP*, *UP*1, and *UP*2) (**c**); middle finger reconstructed with *M* LED (**d**); index, ring, and little fingers reconstructed using the properties of *M* LED (**e**); thumb reconstructed with *T* LED.

A simple cylindrical model is used to render the hand in 3D, with the knowledge of the positional information of five LEDs and the orientation of the palm plane and fingers (Figure 8). It is evident that at least three LEDs are required to reconstruct a plane; using the developed multiple points tracking algorithm, as seen from the figures, we are able to reconstruct the hand with the minimum number of LEDs. Figure 8a depicts the calculated 3D LED locations. From *LP*, *UP*1, and *UP*2 LED locations, the palm plane is reconstructed (Figure 8b). The middle finger location is used to reconstruct the middle finger and index, the ring finger, and the little fingers as well (Figure 8c,d). The thumb is reconstructed with the location information of the thumb LED (Figure 8e).

### 3.7. Gesture Recognition

As an application of the novel multiple points tracking algorithm with merely five LEDs placed on the key points of the hand, a machine learning-based gesture recognition method is formulated. In principle, a simple gesture performed by the hand in front of the LSS sensors is expressed as a vector containing the sequence of LED positions indexed by time. Due to the new approach provided by the LSS and the novelty of the capturing itself, a simple dataset is collected to assess the performance in view of future analysis and improvements with more complex gestures. At present, the number of gestures–classes is fixed as *k* = 6*.* The vocabulary of gestures is given in Table 2.

The gestures are performed at different distances from the sensors using the setup given in Figure 5a. The user is told to perform each of the combinations of gestures at least once during the data collection. The training dataset is then structured involving 10 people, with a total of 600 gestures taken. The feature extraction consists of a procedure to gather the principal characteristics of a gesture to represent it as a unique vector that is able to describe it. A frame-based descriptor approach is used for the same [50,51]. As illustrated in [50], the frame-based descriptor is well suited for extracting features from inertial sensors. However, in contrast to the descriptor created using inertial sensors, in this approach, it is applied to a dataset of different nature that consists of trajectories of the absolute positions indexed by time provided by the developed multiple points tracking algorithm. 

Based on an experimental analysis, the number of captures to describe a gesture is set as 50 captures at 20 fps, where each capture refers to the 3D positions of five LEDs. This corresponds to a fixed length window of 2*.*5 s for each gesture. Even if the system is capable of a frame rate equal to 40 fps, it is sufficient to represent a gesture by down sampling to 20 fps. The mathematical modeling of a gesture descriptor is well described in the latest work of authors [51]. The dataset is then properly formulated and applied in the training and testing phases of a Random Forest (RF) model [52] to validate the performance of gesture recognition.

## 4. Results and Discussion

The effectiveness of the hand tracking algorithm is validated by following experiments: (1) Validation of Rambus LSS, (2) Validation of Multiple Points Tracking, (3) Validation of Latency, and (4) Validation of Gesture Recognition. The algorithms are tested using a computer with IntelCore i5 3.5 GHz CPU, 16 GB RAM.

### 4.1. Validation of Rambus LSS

**Figure 9:**
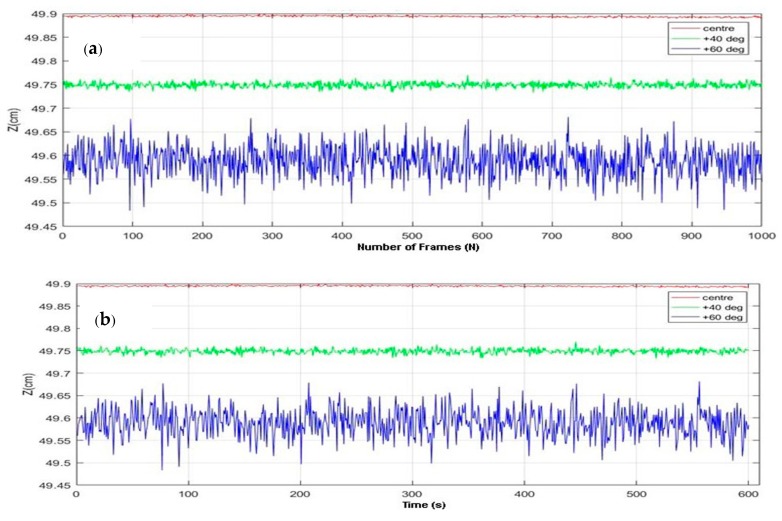
Measured *Z* at different fields of vision (FoVs) (**a**) for 1000 frames (**b**) for 10 min.

The LSSs are fixed on a non-reflective black surface with a spacing of 30 cm (baseline *b =* 30 cm), and the light source is fixed at 50 cm away from the sensor plane (*Z =* 50 cm). The accuracy and precision measurements are done by fixing the light point at five different positions along the experimental plane: along the center of the baseline, on the external sides with a FoV equal to ±40°, and with a FoV equal to ±60°. The root mean square error (RMSE) is used as a measure of accuracy. For evaluating the precision of the sensors, Repeatability (30) and Temporal Noise (31) are used as the standard measures. Then, 1000 frames (*N =* 1000) are taken for each position for the estimation of RMSE and Repeatability. The Temporal Noise is calculated by placing the LED at the same position for 10 min (600 s). In both cases, the *Z* readings (*y_i_*) are plotted. The corresponding plots are shown in Figure 9a,b, respectively. The results are provided in Table 3.

(30) $$Repeatability=\sqrt{\frac{\sum_{i=1}^{N}{\left({\hat{y}}_{i}-\sum_{i=1}^{N}\frac{{\hat{y}}_{i}}{N}\right)}^{2}}{N}}$$

(31) $$Temporal\;Noise=\sqrt{\frac{\sum_{i=1}^{N}{\left[{\hat{y}}_{i}-\left({\hat{y}}_{i}-1\right)\right]}^{2}}{N}}$$

It is inferred from all of the plots that there is no significant variation in either accuracy or precision; the error is low (*≈*0). Precision in terms of Repeatability and Temporal Noise is high for the sensors, which implies consistency in the performance of the LSS technology. The results show that the LSS sensors are able to track the points down to millimeter-level accuracy, which underlines the quality of human hand tracking using these sensors.

### 4.2. Validation of Multiple Points Tracking

**Figure 10:**
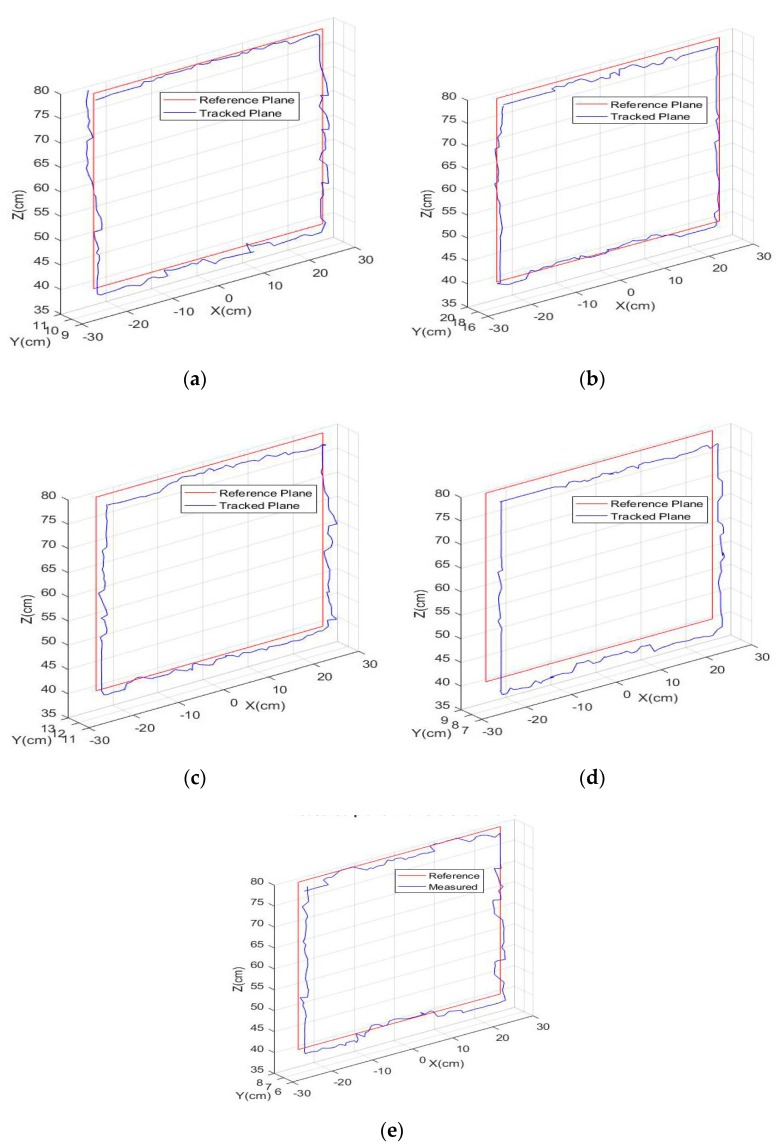
(**a**) *LP*; (**b**) *UP*1; (**c**) *UP*2; (**d**) *M*; (**e**) *T*. Tracked plane with respect to the reference plane for all LED positions.

A wooden hand model with a length of 25 cm, measured from wrist to the tip of the middle finger and a width of 8.3 cm, is used to validate the multiple points tracking accuracy. Using a real hand for these measurements was impractical, because humans cannot maintain their hands in constant positions for the extended periods of time that was required in these experiments. The light points were placed in the exact same positions as described in Section 3.3 for the hand. The wooden hand was then moved onto a rectangular plane in front of the LSSs in the same way as that of single point tracking. The 3D positions of: *LP*, *UP*1, *UP*2, *M,* and *T* referred in Figure 4a were tracked using the developed multiple points tracking algorithm. The extremities of the plane were fixed at −25.2 cm to 25.2 cm laterally along the *X*-axis (which is located at the edges of the 40-deg FoV) with respect to the center axis, and 40 cm to 80 cm longitudinally along the *Z*-axis from the sensor plane. The plane is fixed in such a way as most of the hand movements are in this vicinity. On the horizontal sides, data points are collected for a total of 51 positions, and for the vertical sides, data are collected for a total of 41 positions spaced at 1 cm in order to cover the entire plane. For each point, five raw image frames were acquired by both sensors, and averaged to reduce shot noise. Figure 10 shows the *X*, *Y*, and *Z* measurements of the tracked plane with respect to the reference rectangular plane for all of the five LEDs; namely the *LP*, *UP*1, *UP*2, *M,* and *T* (placed at different locations on the hand). It is remarkable that, for all five positions, the tracked plane is nearly aligning with the reference plane. The corresponding RMSE along each axis and the three-dimensional error using Equation (32) are given in Table 4.

(32) $$Total_{RMSE}=\sqrt{{\left(X_{RMSE}\right)}^{2}+{\left(Y_{RMSE}\right)}^{2}+{\left(Z_{RMSE}\right)}^{2}}$$

**Figure 11:**
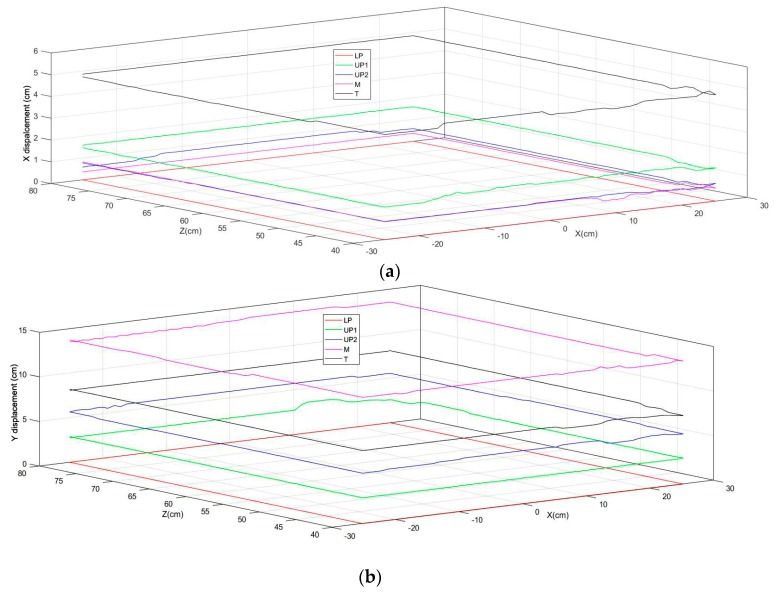
LED positions with respect to LP LED (**a**) *X*-Displacement (**b**) *Y*-Displacement.

For the second experiment, the hand model is moved along the same rectangular plane, using the *LP* LED as the reference, and aligning it with each position. The position of all of the other LEDs was tracked at each point. In each case, the *X* and *Y* displacements of the LEDs with respect to *LP* is found, as shown in Figure 11. It is evident from the plots that the displacement of all of the LED positions along the entire reference plane, along both *X* and *Y* axes, are nearly constant. The deviation for the five positions, with respect to the mean, is computed to validate the results, as shown in Table 5. It can be noted that the mean deviation error is negligible. Using the actual *X* and *Y* displacements from Figure 4b, the displacement error, with respect to *LP* LED positions, is also calculated. The maximum error was recorded for the middle finger LED (*M*), which is maximally displaced with respect to *LP*. The lowest error was recorded for *UP*1, which was the least displaced from *LP*. *UP*2 had a comparable error to that of *UP*1, which is located close to the *UP*1. In all of the cases, the displacement error was less than 1 cm. From the inferences, it can be validated that the relative distance between the LED positions is constant with good accuracy, which implies the quality of the developed hand tracking methodology using the Lensless Smart Sensors. 

The approach developed is robust and able to overcome the difficulty in detecting and distinguishing between the LEDs, i.e., the hand segments, using the image features. On the other hand, the method strongly relies on the accurate and precise ranging of LEDs achieved by the novel sensors. Even if the dynamics of the hand is higher than what the system can cope with—that is, the movement of the fingers is highly non-linear, and LED positions occasionally become close to each other—by modeling in this way, we are able to identify different configurations of the hand.

**Figure 12:**
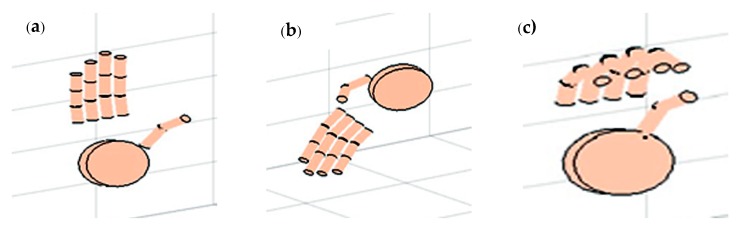
Different orientations of hand in front of LSSs: (**a**) hand held straight; (**b**) hand held upside down at an inclination; (**c**) fingers bend.

**Figure 13:**
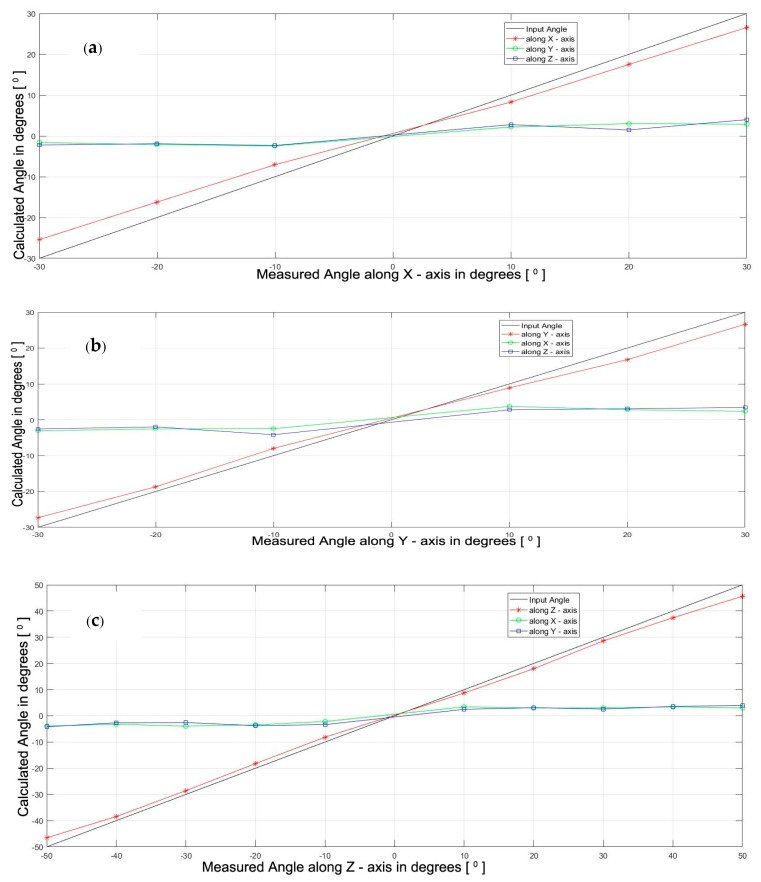

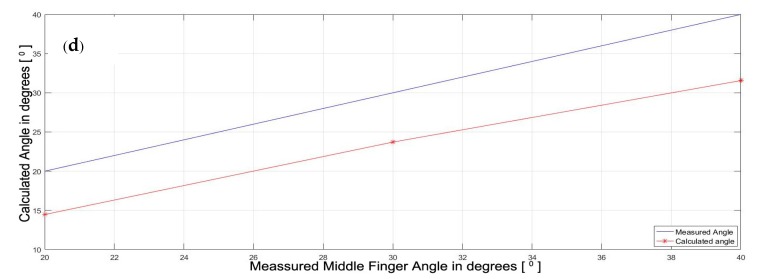
Calculated vs. Actual Orientation: (**a**) Along *X*-Axis; (**b**) Along *Y*-Axis; (**c**) Along *Z*-Axis; (**d**) Along Middle Finger between *S*3 and *d* (*α*).

The orientation estimation is formulated to compute the position and orientation of the middle finger. It is used to approximate the positions and orientations of the remaining fingers, as the simplified model at present has no LEDs fitted on these fingers. The properties obtained for the middle finger are assigned for the index, ring, and little fingers. Some of the other example hand orientations are also shown in Figure 12, which proves that the algorithm is able to recognize different configurations of the hand. The calculated hand orientation angles are shown as a function of the actual hand angles in Figure 13a‒c. The axes of rotation are as given in Figure 7. Along the *Z*-axis, hand orientations are computed from −50° to +50°, whereas along the *X* and *Y* axes, they are calculated only from −30° to +30°, as after this point, no more LEDs are visible in either direction. The finger joint angle between *S*_3_ and *d* (*=α*) is estimated and plotted with respect to the actual angles in Figure 13d. Here also, the possible three readings are plotted, based on the visibility of the LED fitted on the middle finger. The other joint angles are derived from this angle itself, as explained in Section 3.5.3.

Since the light sensors provide only the location information, and there are no other sensors fitted on the hand to measure the exact orientation, it is not expected to be highly accurate, as inferred from the graphs. Since it is difficult to place LEDs on all of the fingers and on all of the phalanges of the fingers because of the occlusions and discriminations investigated in Section 3.2, the algorithm is based on an approximation model, which has its own drawbacks. However, it should be noted that the main objective of this work is to develop a workable system giving emphasis to tracking accuracy rather than orientation. The focus on orientation estimation is to make it simple, yet workable. At this stage, it is impractical to make it much more accurate without using additional sensors that are able to provide angular information. A working demonstrator system can be seen in [53]. In future work, a minimum number of inertial sensors can be considered for integration with the LEDs to estimate all of the orientations of all of the fingers and the related phalanges. Further investigation should be carried out to find out an optimal balance between the number of LEDs and the inertial sensors to achieve accurate tracking, in terms of both the position and orientation, while still considering cost, power, and system complexity.

### 4.3. Validation of Latency Improvements

**Figure 14:**
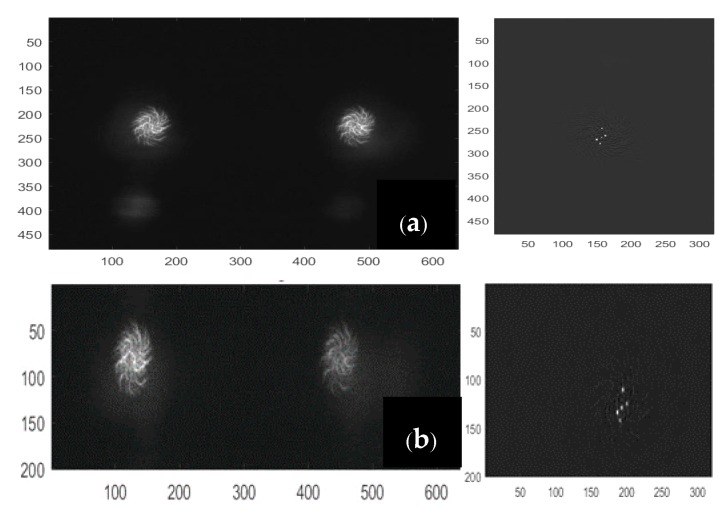
Image Frames and Reconstructed Frames (**a**) before Reducing the Region of Interest (ROI); (**b**) after Reducing the ROI.

In order to reduce latency and facilitate fast hand movement tracking, we have been looking to maximize the frame rate of the data capture, whilst at the same time maintaining a good accuracy and precision in the measurements using the LSS. As discussed in the previous section, frames were averaged during the acquisition stage itself, and consecutively, results averaging two and one frames are compared in terms of the computational time and precision of measurements. The same computer with MATLAB was used for the tests. As a second step, instead of processing the entire image frame, a region of interest (ROI) of reduced size was chosen, and the light points were reconstructed with a PSF of the same size. Specifically, 140 black pixels from the bottom and the top of the image frames, along the *Y*-direction, were removed. It did not negatively affect the diffraction pattern in the acquired frames and PSF, but it did have an impact on the reconstructed frames (Figure 14).

In particular, the time difference between consecutive frames (dt) and estimated frames per second (EFps) are computed for multiple points tracking, as shown in Table 6. The accuracy and precision for the *LP* LED positioned at the center axis of the plane was calculated for the frames of both the full-size and the reduced in size ROI, in the same way as in Section 4.1, by reducing the number of frames to be averaged from five to one using the wooden hand. The results are given in Table 7. As the shot noise is higher, when the number of frames to be averaged is decreased, the accuracy and precision are negatively affected to an extent, but the latency is improved significantly. Although the image quality is degraded, the accuracy is not effected to the same extent because of the implementation of sub-pixel level point detection, which the results confirm. As seen from Table 7, the error keeps on increasing when the FoV is increased, and is varied more at extreme lateral zones. Since the hand tracking and gesture recognition is mostly confined within the region between the two sensors of a baseline *b =* 30 cm, the reduction does not have a significant impact on the application. Therefore, for the final setup, the image frames of the reduced ROI are used without averaging, i.e., one frame/acquisition. This solution led to the achievement of a frame rate of 40 *fps*, which is remarkable about the sensors. It also proves the effectiveness of the proposed tracking algorithm, which does not rely on a complex sensor configuration attached to the hand. The frame rate can be increased further by using high-speed embedded algorithms.

### 4.4. Validation of Gesture Recognition

**Figure 15:**
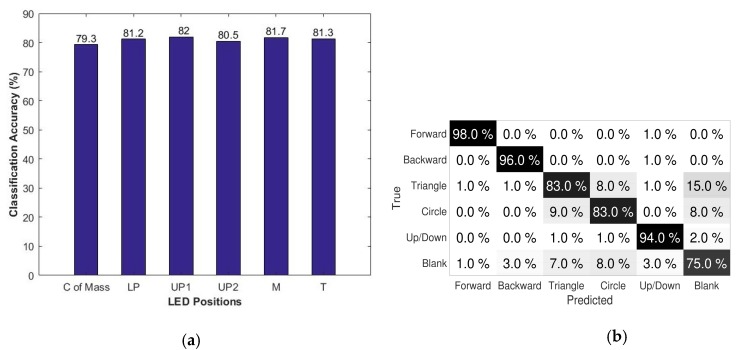
(**a**) Classification accuracy as a function of LED positions; (**b**) confusion matrix for the dataset.

The gesture recognition methodology is employed in a simple painting application where each gesture is associated with a specific task, which can be viewed in [54]. It validated the performance of the Rambus LSS system in gesture recognition applications. A more detailed description of different machine learning approaches and an analysis of the results are provided in [51]. In this work, the classification accuracy as a function of LED position using Random Forest training [52] is evaluated to validate the performance. The graph in Figure 15a shows the classification accuracies of the LEDs at each position on the hand, along with the center of mass. It is noticeable that in all of the cases, the recognition accuracy is greater than 80%, which supports the results. The confusion matrix for the given dataset for the center of mass, considering the five LEDs, for the six gestures given in Table 2 is shown in Figure 15b. It is inferred that the matching accuracy for the predicted gestures with respect to true ones are more than 75% in all of the cases with the limited position and orientation information. This can be further improved by obtaining the information of all of the finger segments, and is able to add more gestures to the dataset.

## 5. Conclusions

In this work, a novel method for positioning multiple 3D points, using the novel Lensless Smart Sensor (LSS), is proposed. It leverages the spatial resolution capabilities of the LSS, and allows for hand movement tracking utilizing vision sensors only. The research activities were focused on hand tracking. It was also extended to a wearable gesture recognition system with high classification accuracy. The location accuracy provided by the proposed hand-tracking algorithm is remarkable, given the type and specifications of the LSS sensors. In spite of having no lens to provide high quality images, it turned out to have advantages for wearable applications when compared to more conventional stereoscopic cameras or the depth sensors. Other techniques using IMUs tend to suffer from drifts, and therefore cannot be used to reliably determine the absolute locations in the world coordinate system. The LSS technology proves to be a promising alternative that is capable of recognizing rotations and translations around both palm and finger coordinates to an extent through the proposed approximation model and tracking algorithms. At present, the palm is considered as a flat plane, but in the future, the model can be calibrated by developing an error function, taking the irregularities of the palm surface into account. In future works, these imaging sensors can be integrated with the inertial sensors in a multimodal configuration, so that shortfalls of individual sensors can be complemented. A constrained biomechanical model of the hand is also considered as an extension of the work. At present, a workable system is developed that is considerably fast at tracking, but in the future, high-speed embedded algorithms will replace the current implementation, and further decrease the latency. Thus, an optimal solution can be achieved that can be suitable for a wearable platform in terms of speed, accuracy, size, and power consumption. This innovative technology can be integrated into a wearable full hand–finger tracking head-mounted system for nearly infrastructure-less virtual reality applications.

## Figures and Tables

**Table 1:** Palm relative distances.

	LP	UP1	UP2
**LP**	*d*_*LP*, *LP*_	*d*_*LP*, *UP*1_	*d*_*LP*, *UP*2_
**UP1**	*d*_*UP*1, *LP*_	*d*_*UP*1, *UP*1_	*d*_*UP*1, *UP*2_
**UP2**	*d*_*UP*2, *LP*_	*d*_*UP*2, *UP*1_	*d*_*UP*2, *UP*2_

**Table 2:** Vocabulary of gestures.

Gesture Label	Gesture Description
0 – Forward	Forward Movement along *Z* axis
1—Backward	Backward Movement along *Z* axis
2—Triangle	Triangle performed on *X*-*Y* plane: Basis parallel to the *X* axis
3—Circle	Circle performed on *X*-*Y* plane
4—Line Up → Down	Line Up/ Down direction on *X*-*Y* plane
5—Blank	None of the previous gestures

**Table 3:** Accuracy and precision of the Rambus Lensless Smart Sensor (LSS).

Precision	Centre	+40 deg	−40 deg	+60 deg	−60 deg
RMSE (cm)	0.2059	0.2511	0.2740	0.3572	0.4128
Repeatability (cm)	0.0016	0.0058	0.0054	0.0210	0.0313
Temporal Noise (cm)	0.0027	0.0082	0.0078	0.0435	0.0108

**Table 4:** Comparison of accuracies for each LED position.

RMSE (cm)	LP	UP1	UP2	M	T
X	0.5054	0.6344	0.5325	0.7556	0.7450
Y	0.3622	0.3467	0.5541	0.9934	0.5222
Z	0.8510	1.0789	0.9498	1.2081	0.7903
Total	1.0540	1.2987	1.1457	1.7370	1.2051

**Table 5:** Displacement error for each LED position with respect to LP LED and mean.

RMSE (cm)	*X*-Axis		*Y*-Axis	
	With Respect to LP	With Respect to Mean	With Respect to LP	With Respect to Mean
UP1	0.5054	0.6344	0.5325	0.7556
UP2	0.3622	0.3467	0.5541	0.9934
M	0.8510	1.0789	0.9498	1.2081
T	1.0540	1.2987	1.1457	1.7370

**Table 6:** Execution time for full and reduced ROI.

No. of Frames Averaged	Full Image Frames (480 × 320)		Image Frames with Reduced ROI (200 × 320)	
	Dt (s)	EFPs	Dt (s)	EFPs
5	0.0553 ± 0.0086	≈ 18	0.0482 ± 0.0134	≈ 21
2	0.0439 ± 0.0182	≈ 23	0.0296 ± 0.0085	≈ 34
1	0.0368 ± 0.0036	≈ 27	0.0248 ± 0.0047	≈ 40

**Table 7:** Accuracy and precision for LP LED at the center.

No. of Frames Averaged	Full Image Frames (480 × 320)			Image Frames with Reduced ROI (200 × 320)		
	RMSE (cm)	Repeatability (cm)	Temporal Noise (cm)	RMSE (cm)	Repeatability (cm)	Temporal Noise (cm)
5	0.4814	0.0025	0.0031	0.5071	0.0018	0.0036
2	0.5333	0.0047	0.0052	0.5524	0.0041	0.0049
1	0.5861	0.0086	0.0074	0.6143	0.0088	0.0091
